# High Residual Gradient Following a Self-Expandable Transcatheter Aortic Valve-in-Valve Implantation — Risk Factor Analysis, Outcomes, and Survival

**DOI:** 10.21470/1678-9741-2020-0424

**Published:** 2022

**Authors:** Tomasz Stankowski, Sleiman Sebastian Aboul-Hassan, Piotr Stepinski, Tomasz Gasior, Mohammed Salem, Temirlan Erkenov, Volker Herwig, Axel Harnath, Anja Muehle, Michel Pompeu B O Sá, Dirk Fritzsche, Bartlomiej Perek

**Affiliations:** 1 Department of Cardiac Surgery, Sana Heart Center Cottbus, Cottbus, Germany.; 2 Department of Cardiac Surgery, Medinet Heart Center Ltd., Nowa Sol, Poland.; 3 Department of Cardiac Surgery, Lodz Medical University, Lodz, Poland.; 4 Division of Cardiology and Structural Heart Diseases, Medical University of Silesia, Katowice, Poland.; 5 Department of Cardiology, Carl-Thiem-Klinikum, Cottbus, Germany.; 6 Division of Cardiovascular Surgery, Pronto-Socorro Cardiológico de Pernambuco (PROCAPE), Universidade de Pernambuco, Recife, Pernambuco, Brazil.; 7 Department of Cardiac Surgery and Transplantology, Poznan University of Medical Sciences, Poznan, Poland.

**Keywords:** Hear Valve Prosthesis, Bioprosthesis, Survival Rate, Myocardial Infarctation, Risk Factors, Survivors

## Abstract

**Introduction:**

Transcatheter aortic valve-in-valve implantation (TAVI-ViV) can be associated with unfavorable hemodynamic outcomes. This study aimed to estimate the prevalence, identify the risk factors, and evaluate the outcomes and survival of patients with high residual gradients after TAVI-ViV.

**Methods:**

A total of 85 patients were included in the study. The cohort was divided into group A, with postprocedural mean pressure gradient (PG) ≥ 20 mmHg, and group B, with mean PG < 20 mmHg.

**Results:**

Postprocedural PG ≥ 20 mmHg was observed in 24.7% of the patients. In a univariate analysis, preoperative gradient, pre-existing patient-prosthesis mismatch (PPM), deep valve implantation, small degenerated valves, and an older generation of transcatheter aortic valves were found to be risk factors for high residual gradient. Multivariate analysis showed that preexisting maxPG > 60 mmHg, implantation level of 4 mm below neo-annulus, and degenerated valve size ≤ 23 mm were independent predictors of high residual gradient. There were no differences in early morbidity (myocardial infarction, pacemaker implantation, stroke, acute renal insufficiency) between groups. Kaplan-Meier estimated that the survival rate was comparable at one and five years regardless of postoperative gradient. Survivors with high residual mean gradient were significantly affected by a high New York Heart Association (NYHA) class.

**Conclusion:**

High residual transvalvular gradient after TAVI-ViV is not rare, but it does not significantly affect mortality. High residual mean gradient has a negative impact on NYHA functional class improvement after the procedure. High preoperative gradient, implantation level, and small failed bioprosthesis may predispose to increased residual gradient.

**Table:** Abbreviations, acronyms & symbols

AF	= Atrial fibrillation
BMI	= Body mass index
BSA	= Body surface area
CABG	= Coronary artery bypass grafting
CAD	= Coronary artery disease
CI	= Confidence interval
COPD	= Chronic obstructive pulmonary disease
DM	= Diabetes mellitus
EF	= Ejection fraction
EOA	= Effective orifice area
EuroSCORE	= European System for Cardiac Operative Risk Evaluation
ICU	= Intensive care unit
LA	= Left atrial
LVEF	= Left ventricular ejection fraction
LVIDd	= Left ventricular internal dimension in diastole
LVIDs	= Left ventricular internal dimension in systole
MODS	= Multiple organ dysfunction syndrome
MV	= Mitral valve
NYHA	= New York Heart Association
OR	= Odds ratio
PAP	= Pulmonary artery pressure
PCI	= Percutaneous coronary intervention
PG	= Pressure gradient
PPM	= Patient-prosthesis mismatch
PVD	= Peripheral vascular disease
SD	= Standard deviation
SPAP	= Systolic pulmonary artery pressure
STS	= Society of Thoracic Surgeons
TAVI-ViV	= Transcatheter aortic valve-in-valve implantation
TIA	= Transient ischemic attack
TV	= Tricuspid valve
USA	= United States of America
VIVID	= Valve-in-Valve International Data

## INTRODUCTION

Transcatheter treatment of failed aortic bioprosthesis has emerged as a safe and effective therapy and is a less invasive approach than chest reopening surgery, especially in patients with prohibitive or high surgical reoperative risk^[1-4]^. Due to the old valve being left behind, transcatheter aortic valve-in-valve implantation (TAVI-ViV) can be associated with unfavorable hemodynamic outcomes, such as increased transprosthetic gradients^[5-7]^. The updated Valve Academic Research Consortium (or VARC-2) recognizes postprocedural transvalvular mean gradient > 20 mmHg as prosthetic valve dysfunction^[8]^. However, the influence of high residual gradient following TAVI-ViV on post-implantation outcomes remains controversial and long-term consequences of higher gradient seem to be unclear^[9-11]^. This study aimed to identify risk factors and evaluate the outcomes and survival of patients with high residual gradients after TAVI-ViV.

## METHODS

### Design and Population

We retrospectively analyzed 86 consecutive high-risk patients with degenerated aortic bioprostheses who underwent TAVI-ViV between March 2010 and July 2019 at Sana Heart Center in Cottbus, Germany. All patients were discussed by the Heart Team consisting of a cardiac surgeon, an interventional cardiologist, and cardiac anesthesiologists and they were disqualified from a conventional repeat surgery due to a high-risk profile. Individuals with acute endocarditis, requiring concomitant cardiac procedures, with previously implanted mechanical or transcatheter valves were excluded. One patient was excluded due to the intraoperative death caused by a perforation of the left ventricle by the guidewire. Finally, 85 patients met the eligibility criteria and were included in the study. The cohort was divided into two groups — group A, with postprocedural mean pressure gradient (PG) ≥ 20 mmHg, and group B, with mean PG < 20 mmHg. The protocol for this study was approved by an institutional review board (ethics committee approval number S-17(dB)/2020) and an individual patient consent was not required due to the retrospective nature of this investigation.

### Data Collection and Outcomes

Our Heart Team, which consisted of a cardiac surgeon, a cardiologist, and an anesthesiologist, always carefully analyzed the treatment strategy. The size of the valves was chosen after an analysis of the multi-slice computed tomography with dedicated OsiriX imaging software (Pixmeo, Geneva, Switzerland). All procedures were performed using the balloon-expandable Medtronic valves (Medtronic, Minneapolis, Minnesota, United States of America [USA]). All demographics, preoperative clinical data, procedural and in-hospital postprocedural data, echocardiography at discharge data, and phone interview follow-up were collected to build the database. Pre-discharge transthoracic echocardiography was performed on the postoperative days 4-13. A complete follow-up was performed mainly by family physicians with a few interviews conducted by phone, with a mean period of 3.8 years (2.1 month to 9.6 years). The primary aim of this investigation was to identify the risk factors of high residual gradient and investigate the effect of the residual transaortic gradient on the overall survival, New York Heart Association (NYHA) functional class, and freedom from aortic valve re-intervention. Secondary objectives were an evaluation of the following: 30-day mortality, early complications (< 30 postoperative days), echocardiographic outcomes (aortic regurgitation, left ventricular ejection fraction), and intensive care unit (ICU) as well as in-hospital length of stay.

### Statistical Analysis

Continuous variables were expressed as means ± standard deviation, while categorical variables were expressed as number and percentages. For continuous data, the Student’s *t*-test or Mann-Whitney’s U-test were used for between groups comparisons, while categorical variables were compared with Pearson χ^2^ test. To identify the independent predictors of high residual gradient (meanPG ≥ 20 mmHg) after TAVI-ViV, we built a multivariate logistic regression model for the whole cohort by using all preoperative variables presented in Table 1 in addition to intraoperative indices such as implantation height or valve type (CoreValve™ or Evolut™ R). A multivariate logistic regression analysis was performed using a stepwise backward regression including only factors identified during the univariate analysis with a *P*-value ≤ 1. Survival curves were calculated using the Kaplan-Meier estimator and the comparison between both groups was made using the log-rank test (Mantel-Cox test). Statistical significance was assumed at *P*<0.05. The statistical analysis was computed with STATISTICA ver. 13 for Windows software (TIBCO StatSoft, Inc., Tulsa, Oklahoma, USA).

**Table 1:** Patients’ demographic, clinical, and echocardiographic characteristics.

Clinical characteristics*	All patients (n=85)	Group A (meanPG ≥ 20 mmHg) (n=21)	Group B (meanPG < 20 mmHg) (n=64)	*P*-value**
Age ± SD, years	79.8 ± 5.7	78.2 ± 6.3	80.4 ± 5.3	0.128
Males (%)	39 (45.9)	8 (38.1)	31 (48.4)	0.409
BSA ± SD	1.86 ± 0.21	1.88 ± 0.20	1.85 ± 0.22	0.578
BMI ± SD	27.3 ± 4.7	28.8 ± 5.1	26.7 ± 4.5	0.075
STS score %, ± SD	12.8 ± 11.1	12.7 ± 11.6	12.9 ± 9.7	0.955
EuroSCORE II %, ± SD	11.8.3 ± 6.0	11.6 ± 4.9	12.2 ± 6.6	0.136
Pre-existing PPM (%)^a^				
None (%)	30 (35.3)	3 (14.3)	27 (42.2)	0.020
Moderate (%)	48 (56.5)	16 (76.2)	32 (50.0)	0.035
Severe (%)	7 (8.3)	2 (9.5)	5 (7.8)	0.804
Preoperative NYHA Class III or IV	74 (87.1)	18 (85.7)	56 (87.5)	0.833
CAD (%)	52 (61.2)	14 (66.7)	38 (59.4)	0.552
Previous PCI (%)	18 (21.2)	5 (23.8)	13 (20.3)	0.734
Previous CABG (%)	30 (35.3)	10 (47.6)	20 (31.3)	0.173
Previous cardiac surgery > 1 (%)	6 (7.1)	2 (9.5)	4 (6.3)	0.611
Previous pacemaker (%)	22 (25.9)	3 (14.3)	19 (29.7)	0.162
Atrial fibrillation (%)	41 (48.2)	7 (33.3)	34 (53.1)	0.115
TIA (%)	2 (2.4)	1 (4.8)	1 (1.6)	0.401
Stroke (%)	10 (11.8)	4 (19.0)	6 (9.4)	0.233
PVD (%)	15 (17.6)	1 (4.8)	14 (21.8)	0.074
Carotid stenosis > 50% (%)	7 (8.2)	2 (9.5)	5 (7.8)	0.804
Pulmonary hypertension (moderate or severe) (%)^b^	8 (9.4)	1 (4.8)	7 (10.9)	0.400
Mean creatinine ± SD, µmol/l	126.3 ± 89.4	115.9 ± 66.3	129.7 ± 96.8	0.494
Chronic kidney disease stage ≥ 3 (%)^c^	58 (68.2)	12 (57.1)	46 (71.8)	0.208
Previous dialysis (%)	3 (3.5)	1 (4.8)	2 (3.1)	0.724
COPD (%)	14 (16.5)	4 (19.0)	10 (15.6)	0.714
Active smoker/ex-smoker (%)^d^	6 (7.1)/11 (12.9)	1 (4.8)/4 (19.0)	5 (7.8)/7 (10.9)	0.636/0.337
Arterial hypertension (%)	80 (94.1)	19 (90.5)	61 (95.3)	0.414
Diabetes mellitus (%)	30 (35.3)	9 (42.9)	21 (32.8)	0.403
Insulin-dependent diabetes mellitus (%)	13 (15.3)	6 (28.6)	7 (10.9)	0.051
Hyperlipoproteinemia (%)	61 (71.8)	16 (76.2)	45 (70.3)	0.604
Elective procedure (%)^e^	73 (85.9)	18 (85.7)	55 (85.9)	0.980
Urgent procedure (%)^f^	7 (8.2)	1 (4.8)	6 (9.4)	0.505
Emergency procedure (%)^g^	5 (5.9)	2 (9.5)	3 (4.7)	0.414
Preoperative intubated (%)	2 (2.4)	0 (0)	2 (3.1)	0.412
Time to failure ± SD, years	9.8 ± 4.1	8.8 ± 3.8	10.1 ± 4.2	0.200
Degenerated valve ≤ 23 mm (%)	62 (72.9)	20 (95.2)	42 (65.6)	0.008
**Type and size of failing surgical bioprosthesis**				
Stented (%)	68 (80)	20 (95.2)	48 (75)	0.044
Stentless (%)	17 (20)	1 (4.8)	16 (25)	0.044
≤ 20 mm (%)	7 (8.2)	2 (9.5)	5 (7.8)	0.804
>20 ≤ 23 mm (%)	55 (64.7)	18 (85.7)	37 (57.8)	0.020
> 23 mm (%)	23 (27.1)	1 (4.8)	22 (34.4)	0.008
**Preoperative echocardiographic parameters**				
Aortic prosthesis stenosis (%)	29 (34.1)	4 (19.0)	25 (39.1)	0.093
Aortic prosthesis regurgitation (%)	14 (16.5)	1 (4.8)	13 (20.3)	0.095
Aortic prosthesis mixed disease (%)	42 (49.4)	16 (76.2)	26 (40.6)	0.005
Leading stenosis (%)	36 (85.7)	13 (61.9)	23 (35.9)	0.036
Leading regurgitation (%)	6 (14.3)	3 (14.3)	3 (4.69)	0.136
Aortic prosthesis meanPG ± SD, mmHg	38.7 ± 18.0	47.4 ± 17.6	35.8 ± 17.3	0.010
Aortic prosthesis peakPG ± SD, mmHg	66.3 ± 27.8	79.0 ± 25.7	62.1 ± 27.4	0.014
EOA ±SD, cm^2^	0.74 ± 0.30	0.70 ± 0.24	0.76 ± 0.32	0.450
MV stenosis ≥ moderate	1 (1.2)	1 (4.8)	0 (0)	0.079
MV regurgitation ≥ moderate	42 (49.4)	7 (33.3)	35 (54.7)	0.089
TV regurgitation ≥ moderate	24 (28.2)	4 (19.0)	20 (31.25)	0.281
LVIDd ± SD, mm	5.27 ± 0.88	5.21 ± 0.81	5.30 ± 0.91	0.737
LVIDs ± SD, mm	3.75 ± 1.02	3.66 ± 1.00	3.78 ± 1.03	0.557
LA diameter ± SD, cm	4.6 ± 0.8	4.6 ± 0.8	4.6 ± 0.8	0.848
LVEF ± SD (%)	50.7 ± 11.0	53.3 ± 9.2	49.8 ± 11.4	0.215
SPAP ± SD, mmHg	45.5 ± 13.7	42.2 ± 10.8	46.5 ± 46.3	0.226

BMI=body mass index; BSA=body surface area; CABG=coronary artery bypass grafting; CAD=coronary artery disease; COPD=chronic obstructive pulmonary disease; EOA=effective orifice area; EuroSCORE=European System for Cardiac Operative Risk Evaluation; LA=left atrial; LVEF=left ventricular ejection fraction; LVIDd=left ventricular internal dimension in diastole; LVIDs=left ventricular internal dimension in systole; MV=mitral valve; NYHA=New York Heart Association; PCI=percutaneous coronary intervention; PG=pressure gradient; PPM=patient-prosthesis mismatch; PVD=peripheral vascular disease; SD=standard deviation; SPAP=systolic pulmonary artery pressure; STS=Society of Thoracic Surgeons; TIA=transient ischemic attack; TV=tricuspid valve

* Continuous variables are presented as means ± SD, whereas categorical data as numbers (n) with percentages (%)

** *P*-value < 0.05 is considered as of statistical significance

a Severe PPM-indexed EOA < 0.65 cm^2^/m^2^ and moderate PPM-indexed EOA 0.65-0.85 cm^2^/m^2^

b Severity of pulmonary hypertension: mild mean pulmonary artery pressure (PAP) 25-40 mmHg, moderate mean PAP 41-55 mmHg, and severe mean PAP > 55mmHg

c Calculated glomerular filtration rate < 60 mL/min

d Quit smoking more than one year before procedure

e Routine admission for operation

f Intervention or surgery is performed on the current admission for medical reasons and these patients cannot be sent home without a definitive procedure

g Operation before the beginning of the next working day after decision to operate

## RESULTS

### Baseline Characteristics

A total of 85 patients were included in the final analysis (45.9% male, mean age 79.8 ± 5.7 years, European System for Cardiac Operative Risk Evaluation II 11.8 ± 6.0%). All demographics and preoperative clinical data of the study population were summarized in Table 1. During the postprocedural echocardiography on days 4-13 after TAVI-ViV, high residual gradient was observed in 21 patients (24.7%). In patients with higher postoperative meanPG, a history of patient-prosthesis mismatch (PPM) after initial surgery, higher gradients pre TAVI-ViV, and smaller and stented degenerated valves were significantly more prevalent.

### Operative Data

The procedure was performed mainly using the femoral approach (97.6%) and conscious sedation with local anesthesia (91.8%). Pre-dilatation was a standard manner and post-dilatation was required in three patients to get the fully expanded valve. No neurological protection, coronary protection system, or valve fracture were used. All implanted valves were self-expandable, CoreValve™ (38.8%) or CoreValve™ Evolut™ R (61.2%). Repositioning of the valve was possible only in the new generation valve (CoreValve™ Evolut™ R) and was needed approximately in half of these patients. Procedures resulting in higher postprocedural meanPG were performed more often with the older valve type, lasted significantly longer, and the new valve was implanted deeper (Table 2).

**Table 2:** Technical indices.

Clinical characteristics*	All patients (n=85)	Group A (meanPG ≥ 20 mmHg) (n=21)	Group B (meanPG < 20 mmHg) (n=64)	P-value**
Anesthetic management				
General anesthesia (%)	7 (8.2)	1 (4.8)	6 (9.4)	0.505
Conscious sedation with local anesthesia (%)	78 (91.8)	20 (95.2)	58 (90.6)	0.505
Surgical approach				
Femoral (%)	83 (97.6)	20 (95.2)	63 (98.4)	0.401
Apical (%)	1 (1.2)	0 (0)	1 (1.6)	0.564
Subclavian (%)	1 (1.2)	1 (4.8)	0 (0)	0.079
Implanted valve type				
CoreValve™ (%)	33 (38.8)	13 (61.9)	20 (31.3)	0.012
CoreValve™ Evolut R™ (%)	52 (61.2)	8 (38.1)	44 (68.7)	0.012
Valve size				
23 mm (%)	44 (51.8)	14 (66.7)	30 (46.9)	0.115
26 mm (%)	33 (38.8)	7 (33.3)	26 (40.6)	0.552
29 mm (%)	4 (4.7)	0 (0)	4 (6.3)	0.241
31 mm (%)	1 (1.2)	0 (0)	1 (1.6)	0.564
34 mm (%)	3 (3.5)	0 (0)	3 (4.7)	0.312
Pre-dilatation (%)	85 (100)	21 (100)	64 (100)	1.000
Post-dilatation (%)	3 (3.5)	1 (4.8)	2 (3.1)	0.724
Evolut™ R repositioning (%)	24/52 (46.2)	5/8 (62.5)	18/44 (40.9)	0.258
Implantation level				
< 4 mm (%)	67 (78.8)	13 (61.9)	54 (84.4)	0.029
> 4 < 8 mm (%)	18 (21.2)	8 (38.1)	10 (15.6)	0.029
Operative time ± SD, min	53.0 ± 22.1	61.4 ± 28.9	50.3 ± 18.8	0.047
Contrast load ± SD, mL	203.9 ± 80.6	199.6 ± 72.0	205 ± 83.7	0.787
Fluoroscopy time ± SD, min	13.2 ± 7.2	14.3 ± 5.2	12.9 ± 7.7	0.440

PG=pressure gradient; SD=standard deviation

* Continuous variables are presented as means ± SD, whereas categorical data as numbers (n) with percentages (%)

** *P*-value < 0.05 is considered as of statistical significance.

### Risk Factors of High Residual Gradient

**Fig. 1:**
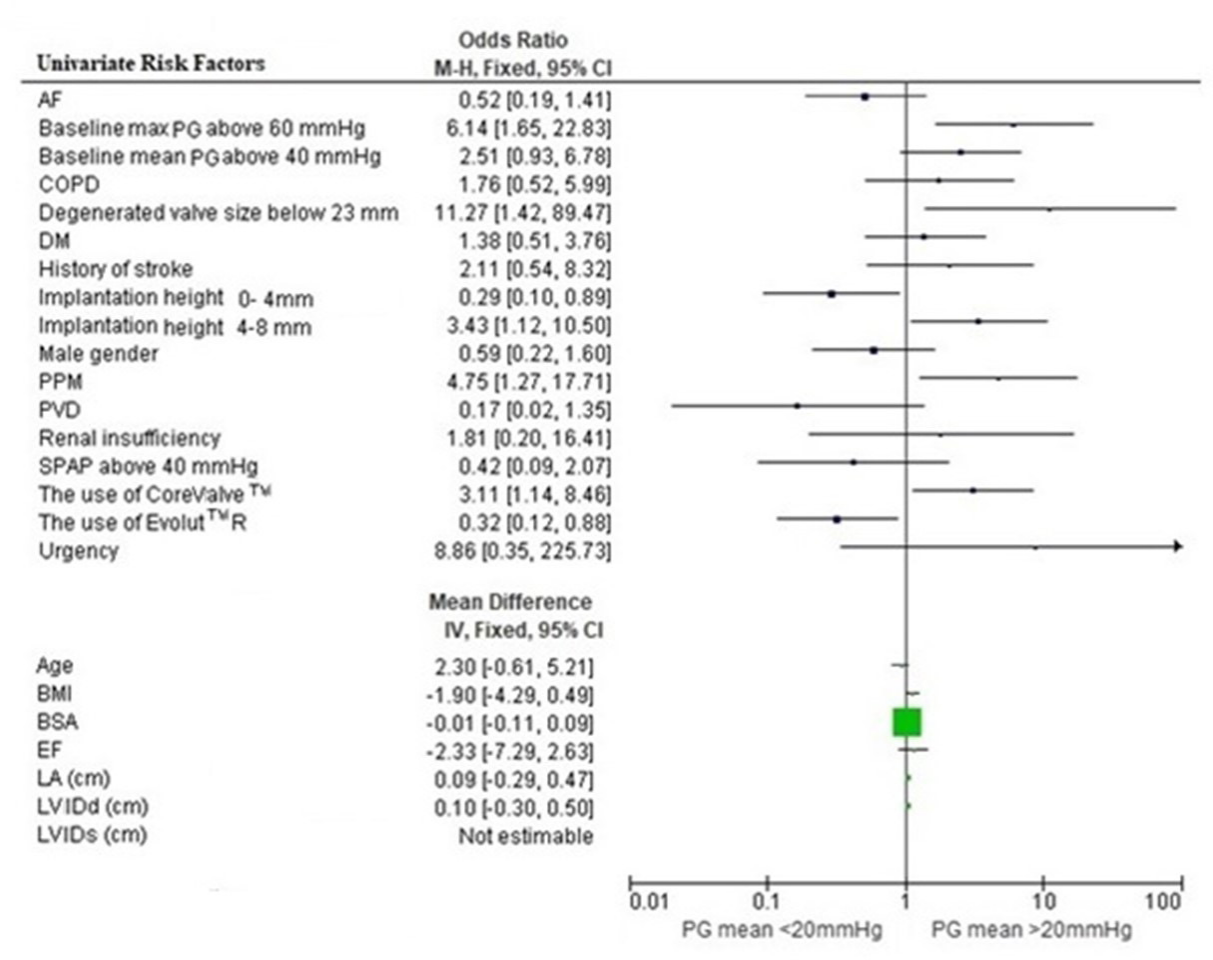
Univariate analysis of factors associated with high residual gradients after transcatheter aortic valve-in-valve implantation (by forest plot representation). AF=atrial fibrillation; BMI=body mass index; BSA=body surface area; CI=confidence interval; COPD=chronic obstructive pulmonary disease; DM=diabetes mellitus; EF=ejection fraction; LA=left atrial; LVIDd=left ventricular internal dimension in diastole; LVIDs=left ventricular internal dimension in systole; PG=pressure gradient; PPM=patient-prosthesis mismatch; PVD=peripheral vascular disease; SPAP=systolic pulmonary artery pressure.

In the univariate analysis, preoperative gradient, pre-existing PPM, deep valve implantation, small degenerated valves, and older generation of transcatheter aortic valves were found to be risk factors for high residual gradient (Figure 1). In the multivariate analysis, preexisting maxPG > 60 mmHg (odds ratio [OR]: 9.3; 95% confidence interval [CI] 1.7-50.9; *P*=0.010), implantation level of 4 mm below neo-annulus (OR: 0.089; 95% CI 0.016-0.494; *P*=0.006), and degenerated valve size ≤ 23 mm (OR: 20.3; 95% CI 1.719-240.2; *P*=0.017) were independent predictors of high residual gradient (Table 3).

**Table 3:** Univariate and multivariate analyses of risk factor for high residual gradient following self-expandable TAVI-ViV.

Clinical characteristics*	OR	Lower 95% CI	Upper 95% CI	*P*-value**
**Univariate Analysis**				
Implantation height 4-8 mm below neo-annulus	3.429	1.120	10.500	0.031
Implantation height 0-4 mm below neo-annulus	0.292	0.095	0.893	0.031
Implanted valve: Evolut™ R	0.322	0.118	0.877	0.027
Implanted valve: Corevalve™	3.106	1.140	8.458	0.027
Pre-valve-in-valve implantation maxPG ≥ 60 mmHg	6.135	1.649	22.831	0.007
Degenerated valve size ≤ 23 mm	11.268	1.419	89.471	0.022
PPM after primary surgery	4.750	1.274	17.709	0.020
**Multivariate analysis**				
Implantation height 0-4 mm below neo-annulus	0.089	0.016	0.494	0.006
Preprocedural maxPG ≥ 60 mmHg	9.308	1.701	50.933	0.010
Degenerated valve size ≤ 23 mm	20.320	1.719	240.210	0.017

CI=confidence interval; OR=odds ratio; PG=pressure gradient; PPM= patient-prosthesis mismatch; TAVI-ViV=transcatheter aortic valve-in-valve implantation

* Continuous variables are presented as means ± SD, whereas categorical data as numbers (n) with percentages (%)

** *P*-value < 0.05 is considered as of statistical significance

### In-hospital Outcomes

Four patients (4.7%) died during the first 30 days after the procedure, one patient in group A and three patients in group B (*P*=0.989). There were no differences in the ICU stay, in-hospital stay, and complication rates between the groups. Kidney function improved after the procedure, regardless of postprocedural mean gradient (Groups A +18.4% *vs*. +14.4% Group B, *P*=0.243). Twenty-six patients (30.6%) had postoperative paravalvular leak, four in group A and 19 in group B. Moderate paravalvular leak occurred in three patients in group B. There were no cases of postprocedural severe paravalvular leak. Early complications (< 30 days post TAVI-ViV) are summarized in Table 4.

**Table 4:** Postoperative outcomes.

Clinical characteristics*	All patients (n=85)	Group A (meanPG ≥ 20 mmHg) (n=21)	Group B (meanPG < 20 mmHg) (n=64)	P-value**
Postoperative echocardiography				
Aortic prosthesis meanPG ± SD (mmHg)	15.6 ± 8.2	26.9 ± 7.0	11.9 ± 4.1	< 0.001
Aortic prosthesis peakPG ± SD (mmHg)	28.5 ± 14.3	46.8 ± 13.6	22.6 ± 8.1	< 0.001
Paravalvular leaks	26 (30.6%)	4 (19.0%)	22 (34.4%)	0.186
Mild	23 (27.1%)	4 (19.0%)	19 (29.7%)	
Moderate	3 (3.5%)	0 (0%)	3 (4.7%)	
Severe	0 (0%)	0 (0%)	0 (0%)	
MV stenosis ≥ moderate	1 (1.2%)	0 (0%)	1 (1.6%)	0.564
MV regurgitation ≥ moderate	26 (30.6%)	5 (23.8%)	21 (32.8%)	0.437
TV regurgitation ≥ moderate	18 (21.2%)	3 (14.3%)	15 (23.4%)	0.373
SPAP ± SD, mmHg	38.9 ± 10.7	40.2 ± 11.3	38.5 ± 10.5	0.561
LVEF ± SD (%)	50.1 ± 12.1	52.5 ± 15.0	49.3 ± 11.0	0.299
Postoperative complications				
30-day mortality	4 (4.7%)	1 (4.8%)	3 (4.7%)	0.989
ICU stay, days	1.4 ± 1.0	1.5 ± 1.1	1.4 ± 1.0	0.661
In-hospital stay, days	6.9 ± 2.0	7.2 ± 2.0	6.7 ± 2.0	0.325
Discharge home	55 (64.7%)	13 (61.9%)	42 (65.6%)	0.757
Postprocedural new dialysis	4 (4.7%)	0 (0%)	4 (6.3%)	0.240
Postprocedural MODS	2 (2.4%)	1 (4.8%)	1 (1.6%)	0.401
Myocardial infarction	0 (0%)	0 (0%)	0 (0%)	1.000
Femoral artery stent graft	6 (7.1%)	1 (4.8%)	5 (7.8%)	0.636
First episode of AF	4 (4.7%)	1 (4.8%)	3 (4.7%)	0.989
Need for pacemaker implantation	4 (4.7%)	2 (9.5%)	2 (3.1%)	0.230
Stroke	4 (4.7%)	1 (4.8%)	3 (4.7%)	0.989
Blood transfusion	6 (7.1%)	1 (4.8%)	5 (7.8%)	0.636
Follow-up period				
Five-year survival	61.7%	62.9%	59.3%	0.938
NYHA class I / II in survivors	50/58 (86.2%)	9/13 (69.2%)	41/45 (91.1%)	0.044

AF=atrial fibrillation; ICU=intensive care units; LVEF=left ventricular ejection fraction; MODS=multiple organ dysfunction syndrome; MV=mitral valve; NYHA=New York Heart Association; PG=pressure gradient; SD=standard deviation; SPAP=systolic pulmonary artery pressure; TV=tricuspid valve

* Continuous variables are presented as means ± SD, whereas categorical data as numbers (n) with percentages (%)

** *P*-value < 0.05 is considered as of statistical significance.

### Follow-up Period

**Fig. 2:**
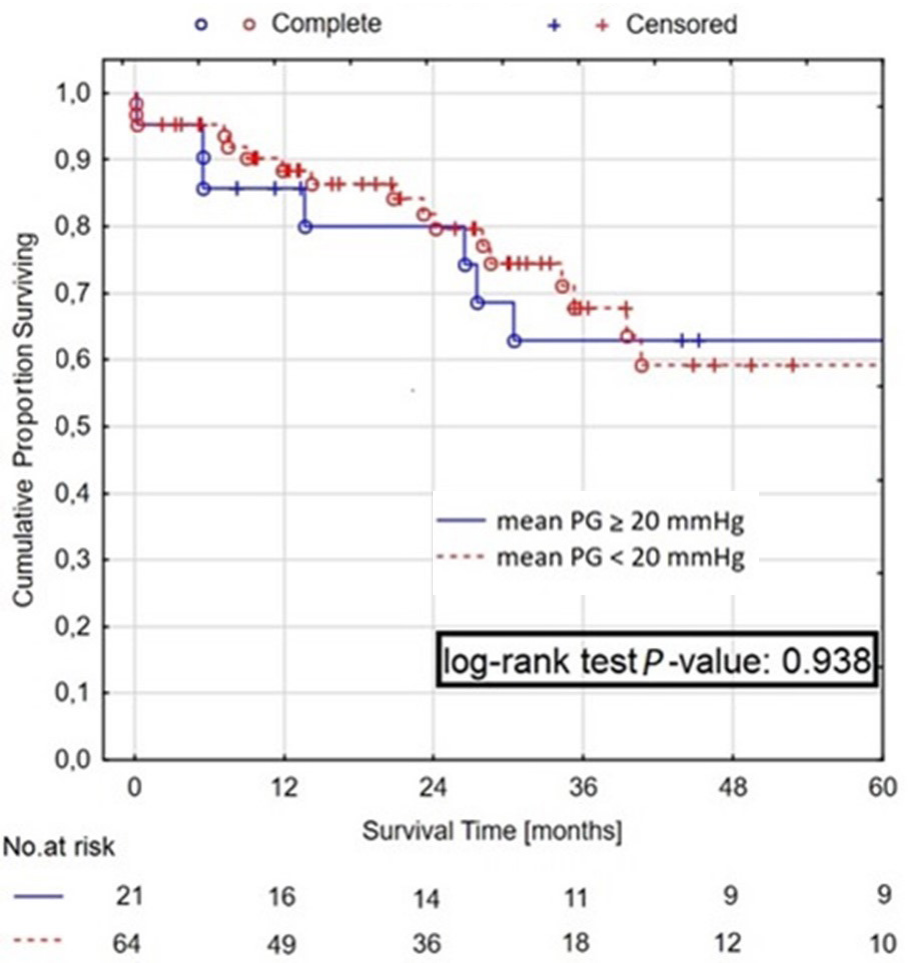
Kaplan-Meier survival curves for all-cause mortality in patient with high residual gradient (mean pressure gradient [PG] ≥ 20 mmHg) and postoperative mean transvalvular aortic gradient < 20 mmHg.

**Fig. 3:**
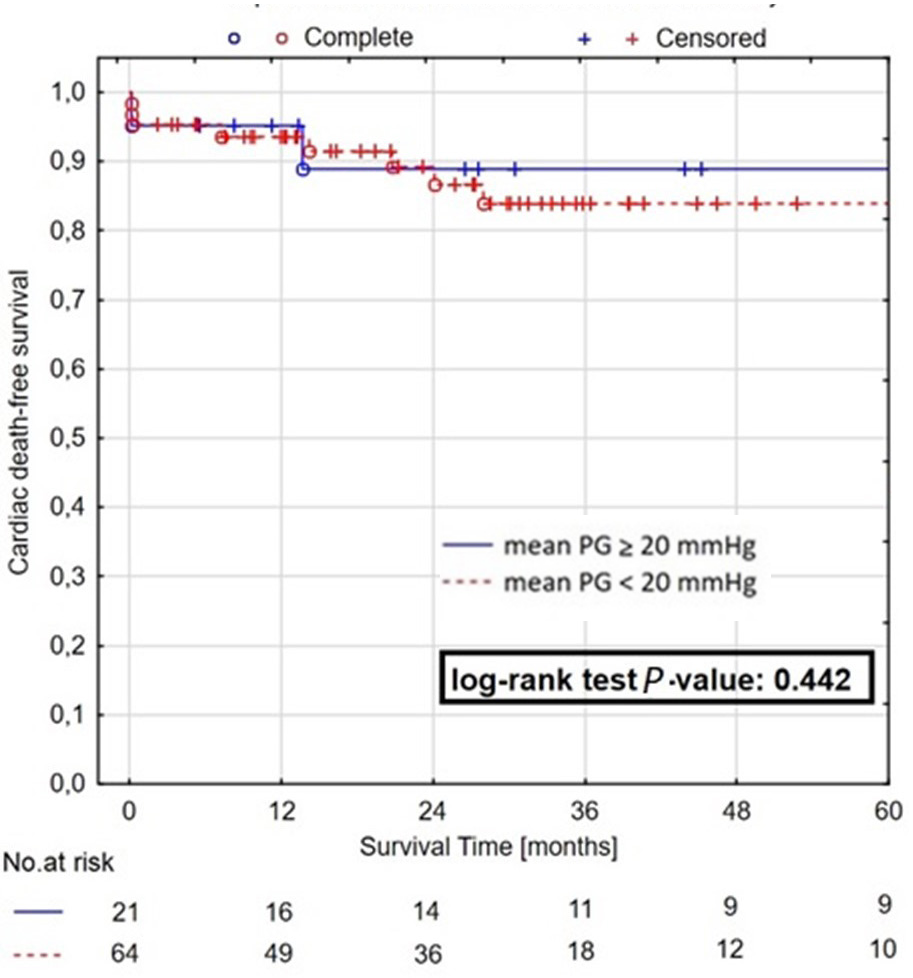
Five-year Kaplan-Meier survival curves for cardiac-related death in patients with postoperative pressure gradient (PG) ≥ 20 mmHg and < 20 mmHg.

Twenty-seven patients (31.8%) died during the study period over nine years. Kaplan-Meier estimated that the survival rate after one, two, and five years was 87.7%, 81.5%, and 61.7%, respectively. The postoperative transaortic gradient had no impact on survival — group A *vs*. group B: one-year survival rate 85.7% *vs*. 88.4%, two-year survival rate 80.0% *vs*. 81.9%, and five-year survival rate 62.9% *vs*. 59.3%, *P*=0.938 (Figure 2). Approximately 40% of deaths (11/27) were caused by a cardiac reason. Two out of eight (25%) deaths in group A and nine out of 19 (47.4%) deaths in group B were cardiac related. Cumulative cardiac death-free survival in one, two, and five years were comparable in both groups — group A *vs*. group B: one-year survival rate 95.2% *vs*. 93.6%, two-year survival rate 88.9% *vs*. 86.7%, and five-year survival rate 88.9% *vs*. 83.9%, *P*=0.442 (Figure 3). At the end of the follow-up period, 50 out of 58 survivors (86.2%) were found in NYHA I or II functional classes (group A 69.2% *vs*. group B 91.1%, *P*=0.044). One survivor with discharge meanPG 30 mmHg was found in NYHA Class IV seven years after TAVI-ViV and required re-TAVI-ViV.

## DISCUSSION

Increased transprosthetic gradients following TAVI-ViV are frequent and range from 17% to 44%, what is similar to our results^[9-14]^. There is some evidence that unfavorable hemodynamic outcomes may impact survival. Pibarot et al.^[15]^ found that patients with pre-existing severe PPM presented more often elevated mean gradient following TAVI-ViV (47.5% *vs*. 29.6%, *P*=0.001). Meanwhile, severe PPM was associated with 2.4- and 1.8-fold higher rates of 30-day mortality and one-year mortality, respectively. Their results are comparable to these presented in the Placement of Aortic Transcatheter Valves 2 valve-in-valve registry. Webb et al.^[9]^ observed the rate of 34.3% patients with high postprocedural residual gradient. They observed a significant higher mortality at one year in patients with postoperative elevated mean gradient (≥ 20 mmHg) (16.7% *vs*. 7.7%, *P*=0.01); however, high gradient was no more a risk factor of all-cause mortality during a three-year follow-up (*P*=0.15)^[1]^. Opposite to these findings, there is substantial evidence suggesting no effect of hemodynamic results on mortality after TAVI-ViV. Akodad et al.^[11]^ compared patients who underwent TAVI-ViV with patients after native transcatheter aortic valve implantation and the repeat procedure was associated with higher postoperative gradient at one month (18.3 *vs*. 11.6 mmHg, *P*=0.0004) and one year (18.1 *vs*. 11.4 mmHg, *P*<0.0001). TAVI-ViV was also associated with a higher rate of patients with mean aortic gradient ≥ 20 mmHg (37.5% *vs*. 8.4%, *P*=0.0002); however, the hemodynamical outcomes do not affect the one-year mortality from a cardiac cause after both procedures (TAVI-ViV 2.1% *vs*. nonTAVI-ViV 2.4%, *P*=0.9). Guimarães et al.^[10]^ analyzed a six-year experience of nine heart centers with TAVI-ViV. Nearly 40% of the patients presented postoperatively high residual gradient > 20 mmHg and they did not observe any significant differences in long-term survival after TAVI-ViV. Authors emphasize the complexity of these group of patients suggesting clinical characteristics and extension of comorbidities as more important in mortality than echocardiographic outcomes. The need of aortic valve re-intervention due to failed bioprosthesis concerns mostly aged patients with a number of comorbidities; therefore, the risk of a repeat open-chest surgery is always significantly increased. Over half of our patients were octogenarians and five patients were over 90 years old. These findings are also confirmed in a paper prepared by Wernly et al.^[16]^. They described the results of 223 patients operated in six German heart centers and observed high postinterventional mean gradient in every fourth patient. Residual stenosis did not affect one-year mortality. Bleiziffer et al.^[17]^ analyzed data from the Valve-in-Valve International Data (VIVID) Registry and observed elevated residual gradients in 27.9% of TAVI-ViV patients. Investigators confirmed no association between high residual gradient and short-term survival. In our study, we did not find any significant differences in one-, two-, and five-year cumulative and cardiac-related mortality rates between the groups. Our results support the opinion that high residual gradient following TAVI-ViV has no impact on mortality in these high-risk profile patients. In our cohort, postoperative gradient did not significantly affect the postoperative survival, which amounted to 87.7% at one year and was similar to the results presented by other authors (Dvir et al.^[13]^ [83.2%], Wernly et al.^[16]^ [76%], Ihlberg et al.^[14]^ [88.1%], and Webb et al.^[9]^ [87.6%]). Notwithstanding, we observed a significantly higher improvement of NYHA functional classes in patients with lower postprocedural mean gradient (*P*=0.044).

Another important goal of our study was to identify risk factors for high residual mean gradient following TAVI-ViV. High preoperative gradient, pre-existing PPM, deep valve implantation, small degenerated valves, and older generation of valves were found to be risk factors for high residual gradient in a univariate analysis. During a multivariate analysis, preexisting peak gradient > 60 mmHg, implantation level of 4 mm below neo-annulus, and the degenerated valve size ≤ 23 mm were identified as independent predictors of elevated mean gradient after TAVI-ViV.

Sá et al.^[18]^ performed a meta-analysis of seventeen studies comprising 71,106 patients (PPM n=25,846 patients and non-PPM n=45,260 patients) and found that more than one third of the patients leave the operation room after native transcatheter aortic valve implantation with significant PPM. Severe PPM was a risk factor of one-year mortality. The same group led by Sá^[19]^ prepared a largest meta-analysis of seventy studies (n=108,182 patients) and found that more than half of the patients after conventional aortic valve replacement present significant PPM direct after the procedure. They observed association between severity of PPM and mortality. Pibarot et al.^[15]^ assessed preexisting severe PPM as a risk factor of high post TAVI-ViV gradients (47.6% *vs*. 29.5%, *P*=0.001). They observed an occurrence of pre-existing PPM in 7.6% of the patients, which is a similar frequency to ours. In our cohort, seven patients (8.2%) presented pre-existing PPM, one patient died eight months after TAVI-ViV, one patient required re-TAVI-ViV, and two survivors were found in NYHA class III. Due to the small number of patients with severe pre-existing PPM, it was difficult to perform a meaningful analysis. Bleiziffer et al.^[17]^ also confirmed a negative impact of PPM on postoperative high gradient after TAVI-ViV.

Scholz et al.^[20]^ focused their study on results after self-expandable TAVI-ViV depending on degenerated valve size. Small valves (< 23 mm) were associated with significant higher postoperative gradient than bigger valves (22.8 ± 9.4 mmHg *vs*. 15.1 ± 7.1 mmHg, *P*=0.013). Simonato et al.^[21]^ used the individuals from the VIVID Registry to study the effect of deep valve implantation on hemodynamical outcomes. They found the strong correlation between deep implantation and better hemodynamics after TAVI-ViV. They defined the optimal implantation level at 0-5 mm below the neo-annulus, which was also noticed in our study. The authors discussed the possibility of aggressive dilatation or even valve fracture, as a novel technique which could ensure the beneficial effect on hemodynamical outcomes. Valve fracture is a new promising technique, which allows receiving the maximum achievable effective orifice area of the new transcatheter valve; however, the currently available evidence is not strong enough to recommend routine valve fracture in TAVI-ViV patients. A large multicenter prospective study evaluating the role of valve fracture on higher postoperative gradients following TAVI-ViV is urgently needed^[21-25]^.

### Limitations

This analysis has several limitations. It is a single-center study with a relatively small sample size. The main limitations of this study are the lack of randomization of the treated groups and the retrospective nature of this investigation. Another limitation is the absence of PPM evaluation following TAVI-ViV and echocardiographic results during the follow-up period. Larger studies will be needed to confirm these results, especially a randomized controlled trial to evaluate the results of intermediate risk patients with degenerated bioprostheses.

## CONCLUSION

Although mean transvalvular aortic gradient ≥ 20 mmHg is not rare after TAVI-ViV procedures, it does not affect significantly either early or late mortality. However, elevated mean gradient reduces the improvement of NYHA functional class after TAVI-VIV. Preoperative gradient > 60 mmHg, deep valve implantation, and small size of the surgically implanted bioprosthesis may predispose to increased residual gradient.
